# “Donor milk banking: Improving the future”. A survey on the operation of the European donor human milk banks

**DOI:** 10.1371/journal.pone.0256435

**Published:** 2021-08-19

**Authors:** Eva Kontopodi, Sertac Arslanoglu, Urszula Bernatowicz-Lojko, Enrico Bertino, Maria Enrica Bettinelli, Rachel Buffin, Tanya Cassidy, Ruurd M. van Elburg, Corina Gebauer, Anne Grovslien, Kasper Hettinga, Ioanna Ioannou, Daniel Klotz, Radmila Mileusnić-Milenović, Guido E. Moro, Jean-Charles Picaud, Bernd Stahl, Gillian Weaver, Johannes B. van Goudoever, Aleksandra Wesolowska

**Affiliations:** 1 Amsterdam UMC, Emma Children’s Hospital, Human Milk Bank, University of Amsterdam, Vrije Universiteit, Amsterdam, The Netherlands; 2 Food Quality and Design Group, Wageningen University & Research, Wageningen, The Netherlands; 3 Division of Neonatology, Department of Pediatrics, Istanbul Medeniyet University, Istanbul, Turkey; 4 Human Milk Bank Foundation, Warsaw, Poland; 5 Department of Midwifery, Centre of Postgraduate Medical Education, Warsaw, Poland; 6 City of Health and Science Hospital, Neonatal Care Unit of the University, Turin, Italy; 7 Maternal and Child Health Unit, Milan, Italy; 8 Neonatal Intensive Care Unit, Hopital de la Croix-Rousse, Hospices Civils de Lyon, Lyon, France; 9 School of Nursing, Psychotherapy, Community Health, Dublin City University, Dublin, Ireland; 10 Abteilung Neonatologie Klinik und Poliklinik für Kinder und Jugendliche, Leipzig, Germany; 11 Neonatal Unit, Milk Bank, Oslo University Hospital, Oslo, Norway; 12 Human Milk Bank, Elena Venizelou Maternity Hospital, Athens, Greece; 13 Center for Pediatrics, Division of Neonatology, Medical Center—University of Freiburg, Faculty of Medicine, University of Freiburg, Freiburg, Germany; 14 Institute of Neonatology, Belgrade, Serbia; 15 Italian Association of Human Milk Banks (AIBLUD), Milan, Italy; 16 CarMeN Unit, INSERM U1060, INRA U1397, Claude Bernard University Lyon 1, Pierre Bénite, France; 17 Department of Chemical Biology & Drug Discovery, Utrecht Institute for Pharmaceutical Sciences, Utrecht University, Utrecht, The Netherlands; 18 International Human Milk Banking Consultant, The Human Milk Foundation, Hertfordshire, United Kingdom; 19 Laboratory of Human Milk and Lactation Research, Regional Human Milk Bank of the Holy Family Hospital, Department of Medical Biology, Faculty of Health Sciences, Medical University of Warsaw, Warsaw, Poland; Federal University of Sergipe, BRAZIL

## Abstract

**Background:**

Provision of donor human milk is handled by established human milk banks that implement all required measures to ensure its safety and quality. Detailed human milk banking guidelines on a European level are currently lacking, while the information available on the actual practices followed by the European human milk banks, remains limited. The aim of this study was to collect detailed data on the actual milk banking practices across Europe with particular emphasis on the practices affecting the safety and quality of donor human milk.

**Materials and methods:**

A web-based questionnaire was developed by the European Milk Bank Association (EMBA) Survey Group, for distribution to the European human milk banks. The questionnaire included 35 questions covering every step from donor recruitment to provision of donor human milk to each recipient. To assess the variation in practices, all responses were then analyzed for each country individually and for all human milk banks together.

**Results:**

A total of 123 human milk banks completed the questionnaire, representing 85% of the European countries that have a milk bank. Both inter- and intra-country variation was documented for most milk banking practices. The highest variability was observed in pasteurization practices, storage and milk screening, both pre- and post-pasteurization.

**Conclusion:**

We show that there is a wide variability in milk banking practices across Europe, including practices that could further improve the efficacy of donor human milk banking. The findings of this study could serve as a tool for a global discussion on the efficacy and development of additional evidence-based guidelines that could further improve those practices.

## Introduction

Human milk banks (HMBs) select, collect, screen, store, process and distribute donor human milk (DHM) that is intended for high-risk infants [1–3]. Since operational safety and quality assurance is considered as a key priority for all HMBs, each practice should be well monitored, and a quality control system should be implemented [1, 4, 5]. Donor recruitment and screening, milk expression, handling and storage (conditions, temperature, duration) both at donors’ homes and in HMBs, transportation to the milk bank (if applicable), bacteriological testing, quality control, pooling, thawing and pasteurization of DHM are included in those practices.

According to the European Milk Bank Association (EMBA), there are currently 248 HMBs located in 26 European countries [6]. Most HMBs operate based on locally implemented standards, nationally or internationally published guidelines. Guidelines published or translated in English are available from the UK, France, Italy, Spain and Sweden. Other countries with nationally recognized guidelines include Germany, Austria, Norway, Slovakia, and Switzerland [1]. HMBs in Poland and Estonia follow internal procedures of conduct that are not subjected to legislation nor are they monitored on a national level. Currently, DHM is not under EU legislation. In Austria, the existing recommendations are legally binding and only in France and Italy federal authorities are closely regulating DHM services [7, 8]. Differences among existing guidelines are mainly due to variations in practices, organization and regulation of HMBs throughout Europe. Those differences include DHM legal classification, location and distribution area of each HMB, and lack of evidence for standardization of some operational points [1, 2, 7].

As no European-wide published guidelines were available, EMBA’s Guideline Working Group was convened in 2015 to undertake this task. Group members from 13 countries (Austria, France, Germany, Italy, Norway, Poland, Portugal, Serbia, Slovenia, Slovakia, Spain, Switzerland, and the UK) completed a detailed survey on the practices followed by their national HMBs. The group investigated whether a consensus on practices was apparent and whether published evidence was available to support recommendations. The EMBA Recommendations for the establishment and operation of human milk banks in Europe became available in 2019 [1]. Notwithstanding the foregoing, and studies on actual procedures in some European countries, a pan-European overview of milk banking practices is lacking and may differ from these recommendations, even among HMBs within individual countries. The aim of the present study was to collect detailed data on the human milk banking practices in Europe, with particular focus on human milk donation, storage, handling, screening and treatment. The outcomes of this study will be used to further strengthen human milk banking guidelines and recommendations.

## Materials and methods

**Fig 1:**
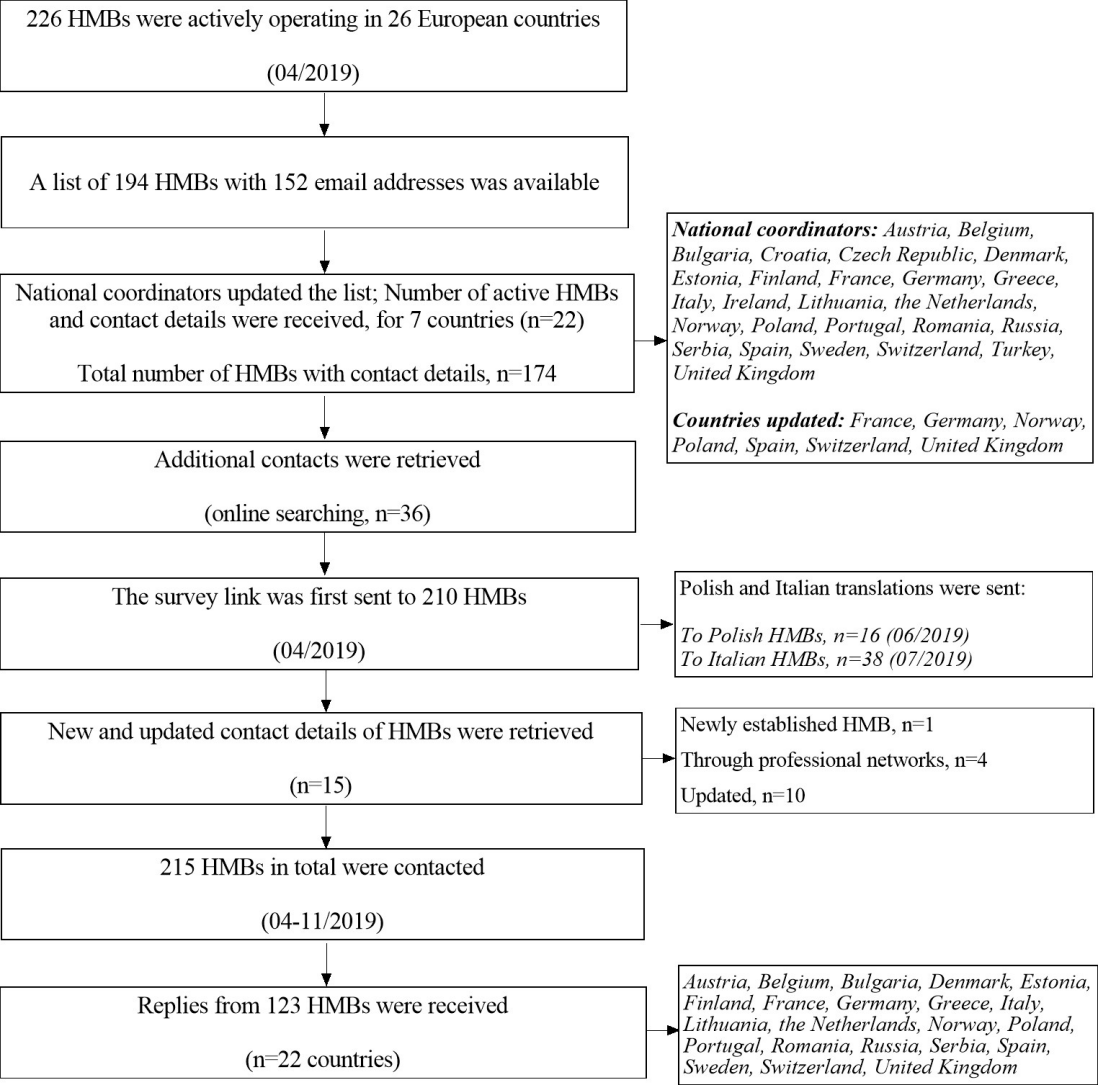
Schematic chart indicating participant flow.

The EMBA Survey Working Group developed a structured web-based questionnaire on milk banking practices, to subsequently distribute to all HMBs that were actively operating in Europe at that time (n = 226, April 2019, EMBA [6]). A list with names and locations of 194 active HMBs in 26 European countries was created, with the joint effort of EMBA and the NGO PATH. Email addresses of 152 HMBs were initially available. The list was then updated and a total number of 215 HMBs with available contact details was finally obtained. Due to a lack of contact details, HMBs in Slovakia and Hungary (n = 11) could not be included in the final list. National coordinators from all 26 countries were appointed, to assist with survey distribution and completion. Their role included updating the number of active HMBs in their own countries, encouraging participation of those HMBs and lastly, minimizing linguistic barriers by offering a native language version of the questionnaire when required (Fig 1).

A general data protection regulation compliant online platform (SurveyMonkey, Portland, USA) was used to facilitate data collection. The selected questions (n = 35) targeted the most critical aspects of the standardized procedures followed in HMBs: donor screening, handling, storage, processing, and microbiological testing of DHM. HMBs had to answer all questions, with the exception of HMBs that do not pasteurize DHM. In that case, HMBs could skip the group of questions regarding pasteurization (n = 7). The Bioethics Committee at Warsaw Medical University reviewed the current study and declared no objection on its conduction (KB/O/23/2021).

A survey invitation email with a web-link to the questionnaire was first sent in April 2019, along with a cover letter explaining the purpose of the study. The letter additionally included detailed information on confidentiality, survey conduction, and contact details of the head of the working group, in case further explanation was needed. Reminders were sent to all participating HMBs in July and August 2019. Next, the authors further contacted all HMBs with incomplete or unclear responses as well as all HMBs with contact details received after July 2019. The survey link remained active until November 2019.

Once the survey was completed, all responses were screened and categorized (per country, per question, per HMB, and as a whole) using Microsoft Excel (2010). GraphPad Prism software 8.0 (GraphPad Inc., La Jolla, CA) was then used for data analysis and visualization. To assess the variation in milk banking practices, adherence to guidelines and extent of milk banking activity, responses for each question were analyzed both for each country separately and for all HMBs together. All calculated percentages were rounded up to the nearest integer. The questionnaire and the list with the responses received per country are available as S1 and S2 Files.

## Results

A total of 123 replies (response rate = 57%) from 22 out of the 26 European countries (85%) were received (S1 File).

### Quality assurance

Most guidelines advise HMBs to implement DHM tracking and tracing systems and to conduct all operational processes based on Hazard Analysis and Critical Control Points (HACCP) and good manufacturing process (GMP) principles [1, 4, 5]. All HMBs implement at least one of the three systems; Approximately 40% of HMBs implement all three aforementioned systems, 30% implement two of the three systems, (HACCP & track and trace 7%, GMP & HACCP 2%, GMP & track and trace 21%) and another 30% only one of the three systems (HACCP 10%, GMP 9%, track and trace 11%).

### Donor screening

The EMBA recommendations state that both verbal interviews and written health questionnaires should be performed as initial donor screening steps [1]. As a second step, all donors should undergo serological screening for a certain panel of diseases [1]. All HMBs indicated that donor selection was based on specific eligibility requirements, although with variation in requirements among the HMBs (Table 1).

**Table 1:** Parameters included in the donor screening processes of European HMBs (n = 123).

	Screening parameters	n (%)
**Lifestyle criteria**	Smoking	121 (98)
	Alcohol	122 (99)
	Drugs of abuse	120 (98)
	Medicines	122 (99)
	HIV risk	116 (94)
	Extreme diets	71 (58)
**Serological screening**	Hepatitis B	123 (100)
	Hepatitis C	123 (100)
	HIV*	123 (100)
	HTLVᶧ	66 (54)
	CMV^¤^	58 (47)
	ALAT/ASAT ratio^†^	9 (7)
	After travelling (specific tests depending on country visited)	46 (37)
	Syphilis	35 (28)
	Chagas disease	6 (5)
	No need to undergo a screening process	3 (2)

*Human immunodeficiency virus

ᶧHuman T-lymphotropic virus

^¤^Cytomegalovirus

^†^Aspartate aminotransferase / alanine aminotransferase.

Some requirements showed very little variation; Lifestyle criteria such as smoking, alcohol, drugs of abuse and medicines, serological screening for human immunodeficiency virus (HIV), hepatitis B and C and the possibility of a donor being HIV infected within a specific period preceding the donation, are included in the donor screening processes of the majority of HMBs (>94%). Nonetheless, extensive inter- and intra-country variation was observed for cytomegalovirus (CMV) and human T-lymphotropic virus **(**HTLV) serological screening, screening for restricted diets (e.g. vegans), aspartate aminotransferase / alanine aminotransferase (ALAT/ASAT) ratio and testing after travelling to specific regions with increased risk of disease transmission.

Out of the 123 participating HMBs, seven HMBs dispense raw DHM only. For that reason, all donors are screened extensively. Serological screening for CMV is performed in six out of those seven HMBs, whereas five perform a serological HTLV screening. However, when adequate pasteurization is performed, CMV screening is not considered necessary [4, 5].

### Start and duration of donation

In 75% of the HMBs, donors are allowed to donate milk from birth onwards while the remaining 25% allows donation only from a specific postnatal week onwards. The maximum duration of milk donation after delivery is specified in 63% of HMBs. A maximum duration of 6 months is set in 26% of HMBs, while 20% of HMBs allow donation for more than 6 months and up to one year (S3 File).

### Expression and storage of human milk at home

Almost all HMBs (99%) provide donors with instructions on how to express, store and handle the milk. Most HMBs (76%) supply the donors with breast pumps for DHM expression (85% electrical, 15% manual).

EMBA’s recently published recommendations state that HMBs should request their donors to freeze DHM as soon as possible, but at least within 24h (48h if collected and stored in a hospital refrigerator) [1]. Our data suggest that 75% of HMBs follow these recommendations. The maximum storage duration of DHM in a domestic freezer before transportation to HMBs varies from 1 week up to 6 months (Table 2).

**Table 2:** Maximum DHM storage duration at home and at the HMB, before and after pasteurization (n = 123).

		Responses	n (%)
**Storage at home**	**Maximum storage duration in a refrigerator (before freezing)**	0h-Immediate freezing	16 (13)
		1h-6h	8 (7)
		12h	9 (7)
		24h	57 (46)
		48h	26 (21)
		72h	3 (2)
		No handling at home	2 (2)
		Other	2 (2)
	**Maximum storage duration in a freezer**	1 week	9 (7)
		2 weeks	19 (15)
		1 month	14 (11)
		2 months	4 (3)
		3 months	24 (20)
		4 months	17 (14)
		6 months	17 (14)
		N/A	6 (5)
		Not specified	5 (4)
		Other	8 (7)
**Storage at the HMB before pasteurization**	**Maximum storage duration in a refrigerator**	0h-Immediate freezing	32 (26)
		12-14h	7 (6)
		24h	35 (28)
		48h	27 (22)
		72h	11 (9)
		Don’t know	5 (4)
		Other	6 (5)
	**Maximum storage duration in a freezer**	1–2 weeks	5 (4)
		1–2 months	14 (11)
		3 months	43 (35)
		4 months	18 (15)
		5 months	2 (2)
		6 months	28 (23)
		> 6 months	2 (2)
		N/A	7 (6)
		Don’t know	3 (2)
		Other	1 (1)
**Storage at the HMB after pasteurization** *	**4 to 5°C**	24h	4 (3)
		48h	1 (1)
		72h	1 (1)
	**-18 to -30°C**	3 months	39 (34)
		4 months	1 (1)
		6 months	58 (50)
		8–9 months	2 (2)
		2 years	1 (1)
		Don’t know	1 (1)
	**-80°C**	1 year	5 (4)
	**Don’t know-N/A**		3 (3)
**Total duration of DHM storage in a freezer (months)** *	**-18 to -30°C**	Minimum	2
		Maximum	49
		Mean ± SD	10.3 **±** 5.61
		Minimum	13.38
	**-80°C**	Maximum	18
		Mean ± SD	15.7 **±** 1.97

*HMBs that do not pasteurize DHM are not included (n = 7). SD, standard deviation.

### Donor human milk handling at human milk banks

Upon arrival at the HMB, DHM should be checked for proper labeling (time of expression and donor identification should be clear) and whether it has remained frozen during transportation [1, 4, 5]. Our data show that about half of the HMBs (52%) have a home collection service, to ensure safe transportation. At the same time, 82% of HMBs check that DHM arriving at the HMB is both frozen and properly labelled. However, 18% of HMBs either accept DHM that arrives already partially thawed or they do not examine the milk’s temperature at all.

In total, 62% of HMBs reported that unpasteurized DHM is kept in a refrigerator for up to 24h awaiting pasteurization or directly stored in a freezer, whereas 22% accept storage in the refrigerator up to 48h and 10% up to 72h. In total, 59% of HMBs set either 3 or 6 months as the maximum storage duration of unpasteurized DHM in the freezer (Table 2).

**Fig 2:**
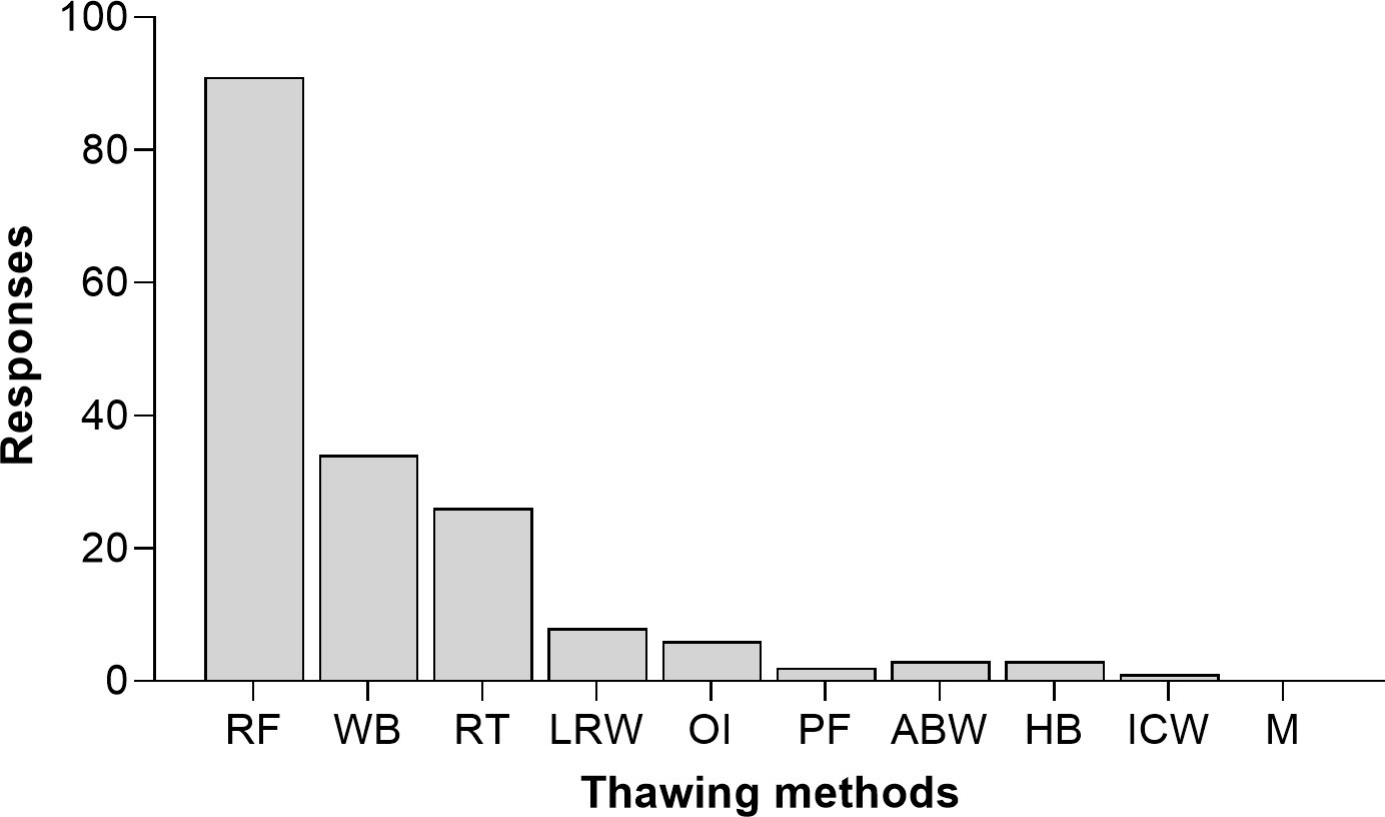
DHM thawing methods (RF = refrigerator, WB = water bath, RT = room temperature, LRW = lukewarm running water, OI = orbital incubator, PF = pasteurizer function, ABW = air bottle warmers, HB = heating blocks, ICW = Immersion in cold water, M = microwave). Answers are presented in absolute values. The participants could select multiple categories, in case multiple thawing methods were included in their practices.

Half of the HMBs (50%) reported that more than one thawing method for DHM is performed. Different methods could be combined due to practical reasons, such as time constraints or variations of the preferred equipment used (e.g. refrigerator, water bath, heating blocks, air bottle warmers). Thawing DHM in a refrigerator is performed in 73% of HMBs, but only half of those HMBs use this method alone. (Fig 2).

Of the HMBs, 26% do not pool DHM, while 54% pool from a single donor and 20% pool from multiple donors (pools of 2–3 donors, n = 11, pools of 4–8 donors, n = 11 and no maximum number of donors specified, n = 2).

### Pre- and post-pasteurization donor human milk screening

There is a large variation in the microbiological screening practices of unpasteurized DHM among HMBs. EMBA recommendations suggest that all pools of milk should be tested before pasteurization, while every batch (referring to the bottles in a single pasteurization cycle) should be tested after pasteurization [1]. Our data suggest that before pasteurization, 23% of HMBs test microbiologically every single container of DHM while 33% test every sample of pooled DHM. Only 2% screen microbiologically both all single and pooled samples of DHM (S1 Fig).

A wide variation was observed in the microbiological criteria defining DHM acceptability before pasteurization (Table 3). In our study, 15% of the HMBs reported either not screening DHM microbiologically before pasteurization or that they are unaware of the criteria applied. DHM with more than 10^6^ Colony-Forming Units (CFU) / ml for total viable bacteria counts (TVC) and 10^4^ CFU/ml for *Staphylococcus aureus* is discarded in 13% of the HMBs, although this number refers to HMBs located in one country only. DHM with TVC>10^4^ CFU/ml is discarded in 9% of HMBs, while in 8% of the HMBs, DHM is discarded when TVC>10^5^ CFU/ml. The NICE guidelines specify that DHM should be discarded if TVC>10^5^ CFU/ml or >10^4^ CFU/ml for *Enterobacteriaceae* or *S*. *aureus*, which is followed by 8% of HMBs [5]. The EMBA recommendations suggest accepting DHM containing ≤10^5^ CFU/ml non-pathogenic organisms and no pathogens for each DHM pool tested before pasteurization [1], which is done by 7% of HMBs. The applied criteria varied greatly, not only between but also within countries. HMBs from only two countries (out of the eight countries that were represented by n >3 HMBs in this study), follow a specific guideline with adherence ≥60% per country.

**Table 3:** Microbiological criteria defining DHM acceptability before pasteurization (n = 123).

Responses	n (%)
Total flora ≤10^2^ CFU/ml	2 (2)
Total flora <10^3^ CFU/ml	3 (2)
Total flora <10⁴ CFU/ml	11 (9)
Total flora <10⁵ CFU/ml	10 (8)
Total flora <10⁵ to <10⁴ CFU/ml	3 (2)
Total flora <10^6^ CFU/ml	6 (5)
Total flora 10^3^−10^5^ CFU/ml, other flora <10^3^ CFU/ml	2 (2)
Total flora <10⁵ CFU/ml, other flora <10^3^ CFU/ml	2 (2)
Total flora <10⁵ CFU/ml, pathogens = 0 CFU/ml	2 (2)
Total flora ≤ 10⁵ CFU/ml, *S*. *aureus* ≤ 10⁴ CFU/ml	2 (2)
Total flora ≤ 10⁵ CFU/ml, *S*. *aureus* and *Enterobacteriaceae* ≤ 10⁴ CFU/ml (NICE guidelines)	10 (8)
Total flora ≤ 10⁵ CFU/ml, *S*. *aureus* and *Enterobacteriaceae* ≤ 10⁴ CFU/ml, *Bacilli* = 0 CFU/ml	2 (2)
Total flora <10⁵ CFU/ml, *S*. *aureus* <10⁴ CFU/ml, *Coliform*s<10^3^ CFU/ml	2 (2)
Total flora ≤ 10^6^ CFU/ml, *S*. *aureus* and *Enterobacteriaceae* ≤ 10⁴ CFU/ml, *Bacilli* = 0 CFU/ml	2 (2)
Total flora <10^6^ CFU/ml, *S*. *aureus* <10⁴ CFU/ml **^a^**	16 (13)
Only when *S*. *aureus* <10⁴ CFU/ml ^b^	9 (7)
Only when pathogens <10⁴ CFU/ml	2 (2)
DHM is assessed by the dornic acid test ^c^	4 (3)
Swedish guidelines ^d^	2 (2)
Not tested/Don’t know	18 (15)
Other ^e^	13 (11)

^a^, ^b^, ^c^ This criterion is applied by HMBs located in one country only (n = 3 countries, one criterion per country)

^d^ refers to the exact response received (acceptance criteria were not specified in detail)

^e^ HMBs with different individual acceptance criteria (n = 13).

Microbiological testing after pasteurization is always performed in 56% of HMBs and regularly in 27%, where regularly includes once a month, every 10 pasteurization cycles, only when there are concerns about the processing, or when new equipment or employees are introduced. Microbiological testing after pasteurization is never performed in 11% of HMBs, while 6% do not pasteurize DHM.

After pasteurization, 62% of HMBs accept only DHM with no detected microbial growth. Pasteurized DHM with TVC≤10 CFU/ml is accepted in 13% of HMBs, while 8% accept DHM with counts ≥100 CFU/ml or have no defined thresholds. The remaining 17% either do not pasteurize DHM (6%) or do not perform microbiological testing after pasteurization (11%).

### Donor human milk treatment

Holder pasteurization (62.5°C for 30 minutes) is recommended for DHM treatment. The ideal process should consist of a rapid heating phase, followed by a phase where the temperature remains constant, and finally a rapid cooling phase [1, 4, 5, 9]. Our findings show that DHM is heat treated in 94% of HMBs. Four HMBs in Norway, two HMBs in Germany and one HMB in Sweden represent the remaining 6% (n = 7) that do not pasteurize DHM. DHM is heated at 62.5°C for 30 minutes in 95% of the HMBs that pasteurize DHM, while slightly different parameters (60–64°C for 30-65min, n = 5 and 75°C for 15sec, n = 1) are applied by the remaining 5%. The majority of HMBs (70%) reported using standard pasteurizers, with water as the heating medium. Shaking water baths and dry heating pasteurizers are lesser used (11% and 11%, respectively) and 8% did not specify pasteurizer design.

The same volume of DHM is included in every bottle within a pasteurization cycle by 66% of the HMBs. Of the remaining 34% of HMBs that pasteurize different DHM volumes within the same cycle, 6% answered that volumes depend on their needs, on available bottle sizes or that they are not aware of the volumes used. Differences in DHM volume ranging from 40ml to 90ml within the same pasteurization cycle were reported by 16% of HMBs and from 100ml to 210ml by 12% of HMBs.

**Fig 3:**
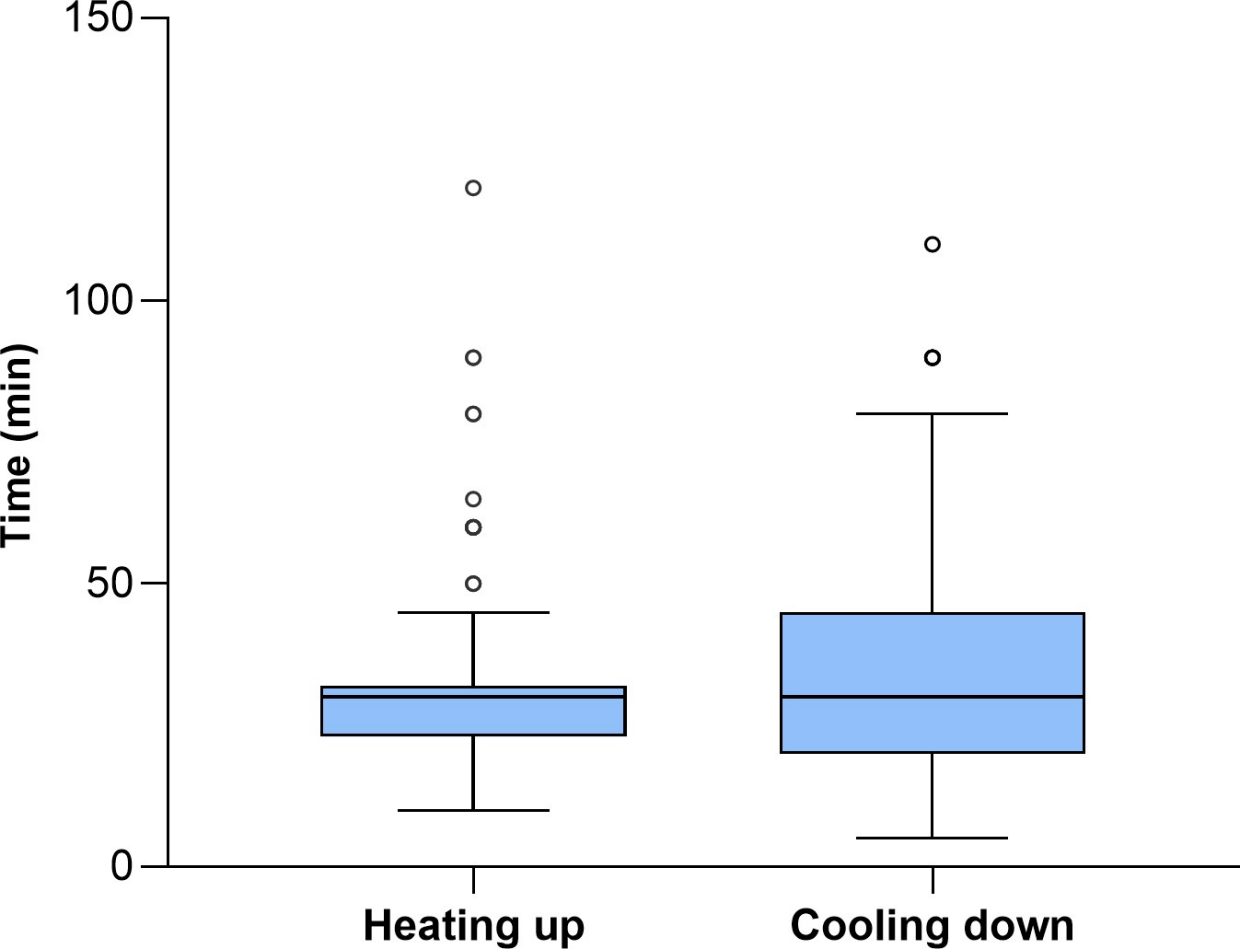
Heating up times to pasteurization temperature (n = 103. “Don’t know”, n = 13) and duration of the cooling cycle (n = 106. “Don’t know/not controlled”, n = 10).

The time required to raise the temperature of DHM to the pasteurization temperature (heating up time) and the cooling down time, which are important factors in processing efficacy, showed large differences among HMBs; Reported durations ranged from 10 to 120 minutes and from 5 to 110 minutes respectively, while the total processing time, which corresponds to the sum of the heating up time, the holding time and the cooling down time, ranged from 20 to 200 minutes (Fig 3). This could be attributed to the combination of different pasteurizer designs, DHM volumes and variations in the execution of the cooling phase. Lastly, 12% of HMBs do not monitor the temperature/time progression during the pasteurization process.

The cooling phase is automatically performed by the pasteurizer in 78% of HMBs and manually in the remaining 22% of HMBs, e.g. with iced water baths (n = 10), freezers (n = 3), refrigerators (n = 4), blast chillers (n = 7) or at room temperature (n = 2). The majority of HMBs (68%) cool DHM to a temperature between 2 and 6°C.

### Post-pasteurization storage

Pasteurized DHM is stored at -18°C to -30°C in 88% of HMBs. Almost all (96%) of those HMBs, store pasteurized DHM for 3 to 6 months while 3% exceeds this storing period. Only 5% keep pasteurized DHM for 1–3 days at a refrigeration temperature (Table 2).

The overall storage duration of DHM in a freezer (in a domestic freezer and in a HMB before and after pasteurization) was largely different among HMBs. The different storage durations applied are shown in Table 2.

## Discussion

Our findings showed a huge variation in the practices currently applied across European HMBs. Diversity of practices was observed not only between but also within countries, indicating that even when national guidelines existed, actual practices differed.

One of those practices was the maximum storage time in a freezer before pasteurization. This reflects the variation in the published recommendations which ranges from 1–12 months [4]. Similarly, on a global level, the regulations established by the National Sanitary Surveillance Agency (ANVISA) in Brazil, indicate 15 days as the recommended maximum DHM storage time at a temperature of -3°C, while the recommendation from the Human Milk Banking Association of North America (HMBANA) is a maximum of 3 months, at -20°C [10, 11]. Prolonged storage duration (>3 months) could enable HMBs to secure adequate DHM supplies and reduce the disposal of expired DHM. However prolonged storage could also impact the quality of DHM. Studies investigating the effect of frozen storage (1–9 months) on specific proteins, report contradictory results (S4 File) [12–23]; Freezing DHM for 3 months at -20°C has been found to cause a minimal loss of its biological activity [12], but a significant decrease in lactoferrin levels has been also reported [13, 18]. On the contrary, one study found no effect on lactoferrin and SIgA levels after 9 months at -20°C [19]. Freezing pasteurized DHM at -20°C for 8 months did not decrease the macronutrient or energy content [20].

In conclusion, storage of DHM at -20°C for a maximum of 3 months seems to be safe without substantial loss of quality of the DHM. Probably a longer storage time can be applied, although more data are needed to make such a recommendation.

After storage of frozen DHM, thawing methods vary among HMBs. This is consistent with the existing recommendations, as not one specific thawing method is currently recommended. Thawing DHM in a refrigerator, in a water bath, at room temperature, under running lukewarm water or with special thawing devices are all methods described in published guidelines (S5 File), thus including both slow and quick thawing methods [1, 5, 24–29]. The Brazilian regulations additionally allow thawing DHM in a microwave, but only when the exposure time for specific DHM volumes has been calculated based on the equipment specifications, size and shape of the bottles, so that DHM temperature does not exceed 5°C. According to the HMBANA guidelines, the DHM temperature while thawing should remain below 7.2°C, while EMBA recommendations specify that DHM temperature should not exceed 8°C [10, 11]. A considerable risk when thawing DHM at room temperature or higher is bacterial growth [4]. When thawing DHM in a water bath or under running water, additional precautions should be taken to avoid submersion and cross-contamination through ingress of water in the event of the containers not being properly sealed [4]. Therefore, we propose that guidelines allowing such methods should extensively describe the monitoring procedure as well as all potential hazards.

Overall, since thawing can affect both the quality and the safety of DHM, certain practices should be preferred. Refrigeration overnight is considered as optimal, as no significant increase in bacterial counts for 24h has been reported [4, 30, 31]. Thawing DHM with waterless defrosting devices could be another option, as the risk of cross-contamination due to contact with water is eliminated while at the same time quicker thawing times are achieved [32]. As such devices can be conveniently used in HMBs, further research is needed in order to conclude on their effects on DHM quality.

Most guidelines recommend pooling of unpasteurized DHM from a single donor only [1, 5, 25, 29]. However, some guidelines also mention that multi-donor pools may be acceptable, but only from a limited number of donors (S5 File) [4, 26, 28]. Multi-donor pools are also allowed in other non-European published guidelines such as the Brazilian and the HMBANA guidelines ([10, 11]). In our study, 25 HMBs from various countries use multi-donor pools. One reason for using multi-donor pool could be the compensation for possible nutritional differences among donors, although nowadays, both nutrient analyses using human milk analyzers and individualized fortification can be performed. Pooling also enables smaller volumes of DHM to be used sooner, thus reducing pre-pasteurization storage times. To avoid microbial contamination and to ensure donor traceability, future guidelines should extensively describe the practices that should be followed if pooling is applied.

For DHM treatment, holder pasteurization is performed in almost all participating HMBs, with the exception of a few HMBs in Germany and Sweden, and the majority of HMBs in Norway. This method effectively inactivates DHM microbial contaminants, but the specific time-temperature combination used may negatively affect the activity of several DHM components [9]. Ensuring rapid heating up and cooling down is also of crucial importance; Since DHM bioactive components start to be significantly damaged from 58°C, the time DHM is heated above this temperature should be limited [9, 33]. In addition, optimized pasteurizers with shorter plateau duration and better temperature control during a cycle have been shown to better preserve SIgA, lactoferrin and lysozyme in DHM [34]. However, no recommendations are currently available regarding the maximum heating up time. Only the Brazilian regulations include detailed information on how to calculate the heating up time, based on the DHM volume, type and number of bottles used. The regulations additionally specify that all bottles should contain the same volume of DHM and the starting temperature should be stable and around 5°C. A table of the calculated heating up times for all different DHM volumes used in the HMB should then be created [11].

In addition, a rapid cooling down would minimize spore germination. To avoid bacterial proliferation, a temperature drop from 62.5°C to 25°C in 10 minutes is suggested [4]. Moreover, a total of 20 minutes to reach a final DHM temperature ≤ 8°C has been recommended [26]. Although temperatures <10°C are mostly suggested [1, 4, 5, 26, 35], no consensus currently exists over time and temperature.

Our data show that DHM is at present exposed to slow heating up and cooling down phases, which is in contrast with the recommended rapid pasteurization performance. The wide range of reported heating up and cooling down times could be due to the different pasteurizer designs, the final cooling temperature, and the differences in DHM volume within one pasteurization cycle. Dry heating pasteurizers seemed to expose DHM to longer total processing times, but as the majority of those pasteurizers do not include an automated cooling down phase, this is mostly dependent on how the cooling phase is performed (S1 Data).

Due to the various practices applied, recommending a single practice would be challenging. However, additional recommendations on pasteurization efficacy can be added to the existing guidelines. A recommendation on the optimal duration of both phases could facilitate the standardization of pasteurization.

Bacteriological screening practices of DHM were quite variable both between and within countries in our study. This is in line with the EMBA’s Guideline Working Group findings, where no consensus could be derived for either the defined criteria or for the frequency of testing [1]. More than half of the HMBs reported testing DHM only regularly (e.g. once a month). Interestingly, stricter practices were not applied even in HMBs performing multi-donor pools, thus increasing the risk of administrating DHM that does not meet the acceptance criteria. EMBA’s recommendations (Test all DHM pools before pasteurization and accept DHM with ≤10^5^ CFU/ml of non-pathogens, test each batch after pasteurization and accept only DHM with no detected microbial growth) could be further adopted in order to increase the safety of the recipients. Regarding donor screening, the recruiting criteria should be flexible and adaptable to country-specific infectious diseases risk factors and the distribution of health-related events worldwide.

## Conclusions

This study investigated actual human milk banking practices among European HMBs, with a high number of participants. Our findings highlight the wide variability covering most human milk banking practices in Europe, especially with regards to the DHM processing and bacteriological screening. When practices were evaluated based on both national and international guidelines, adherence was low, specifically with respect to the application of specific control systems, DHM storage, thawing, processing and screening. However, since variation in certain practices can exist without posing any safety risk, concluding on whether the observed variations have a negative impact on actual DHM quality and safety, remains a high priority. Risk assessment strategies may further assist in evaluating the effect of this variability, while future research may also focus on further analyzing the causes of these variations. More extensive guidelines should therefore become available, while the need for developing guidelines covering all essential steps in DHM handling with large variations in execution such as DHM processing and storage, is of particular importance.

## Supporting information

S1 File Human milk banks contacted and response rate. (PDF)

S2 File Questionnaire. (PDF)

S3 File Postnatal week onwards that the donors are allowed to donate, and the maximum duration of donation. (PDF)

S4 File Effect of frozen storage (-20°C and -80°C) on DHM components, before and after pasteurization. (PDF)

S5 File Published guidelines on storage, thawing, pooling and frequency of bacteriological testing of DHM. (PDF)

S1 Fig Frequency of DHM microbiological testing before pasteurization. Multiple selection of answer options was possible; For all HMBs that selected more than 1 option, combined categories were created. *Combined categories.
(TIF)

S1 Data Dataset. (XLSX)
