# Supplementary material for: “Donor milk banking: Improving the future”. A survey on the operation of the European donor human milk banks
Source: PLoS One. 2021 Aug 19;16(8):e0256435. doi: 10.1371/journal.pone.0256435 (PMC8376009; doi:10.1371/journal.pone.0256435)
Supplement: S1 File — (PDF) [file pone.0256435.s001.pdf]

S1 File. Human milk banks contacted and response rate.

Country	Number of contacted HMBs	Completed responses	Response rate per country	Responses per country/Total responses
France	34	22	65%	18%
Italy	38	15	39%	12%
Germany	22	14	64%	11%
Spain	15	14	93%	11%
Poland	16	13	81%	11%
Norway	12	10	83%	8%
Sweden	28	8	29%	7%
United Kingdom	16	7	44%	6%
Austria	5	2	40%	2%
Belgium	4	2	50%	2%
Greece	2	2	100%	2%
Russia	3	3	100%	2%
Switzerland	7	2	29%	2%
Bulgaria	1	1	100%	1%
Lithuania	2	1	50%	1%
Netherlands	1	1	100%	1%
Estonia	1	1	100%	1%
Finland	1	1	100%	1%
Portugal	1	1	100%	1%
Denmark	2	1	50%	1%
Romania	1	1	100%	1%
Serbia	1	1	100%	1%
Croatia	1	0	0%	0%
Czech Republic	1	0	0%	0%
**Total**	**215**	**123**		
