# Supplementary material for: “Donor milk banking: Improving the future”. A survey on the operation of the European donor human milk banks
Source: PLoS One. 2021 Aug 19;16(8):e0256435. doi: 10.1371/journal.pone.0256435 (PMC8376009; doi:10.1371/journal.pone.0256435)
Supplement: S2 File — (PDF) [file pone.0256435.s002.pdf]

S2 File. Questionnaire. Donor Milk Banking:

Improving the future[1]

This questionnaire is being sent to you on behalf of the European Milk Bank Association.

Storage, handling and heat treatment, play a crucial role in ensuring the quality and safety of banked donor human milk. With your help, this survey will evaluate current practices in an effort to improve the future of milk banking. The survey should take approximately 20 minutes to complete.

All individual responses will be kept strictly confidential and will never be associated with your name. SurveyMonkey.com is a GDPR compliant (General Data Protection Regulation) website.

---

1. **Background information**
    1. Name of the person completing this questionnaire
    2. Name of milk bank, city and country
    3. Year the milk bank operated for the first time
    4. Is the milk bank linked to a hospital? if yes, name the hospital.
    5. If the milk bank supplies external hospitals, please write down the number of external hospitals supplied.
    6. Please write down the total volume of donor milk supplied (to all hospitals), for 2018.

2. **Equipment**
    1. Number of pasteurization machines
    2. Number of refrigerators
    3. Number of freezers
    4. Total number of rooms

3. **Does the milk bank provide donors with breast pumps?**
    - o Yes
    - o No

4. **If yes, how many electrical and how many manual?**

    Use "N/A" (not applicable) if the figures are not known.

    1. Number of electrical pumps
    2. Number of manual pumps

5. **Staff qualification**

    (In your answer please state if full time or part time. If part time, please specify working hours per week)

    1. Staff working in the milk bank
    2. Number of workers with IBCLC (international board-certified lactation consultant)
    3. Number of workers with an academic degree (please specify; e.g. PhD, Asoc.Prof., Prof., etc.)

---

[1] All questions below are displayed as appeared in SurveyMonkey: Multiple check boxes are symbolized with "□" and multiple choice (Yes-No-Don't know answers) with "o". Numbers (1., 2., 3., etc.) are used in this text only, to symbolize the questions with a comment box as an answer option in SurveyMonkey.

4. Number of neonatologists
5. Number of dietitians
6. Others (please specify)

6. Have you completed EMBA's survey in 2011?

- o Yes
- o No
- o Don't Know

7. Please complete the following:

Use "N/A" (not applicable) if the figures are not known.

1. Total volume of milk donated to the bank (L) during 2018
2. Number of donors during 2018 (Jan-Dec)

8. If you are able, please complete the number of breast milk donors for the following years:

Use "N/A" (not applicable), for the years the Milk Bank was not active or if the figures are not known.

1. 2017
2. 2016
3. 2015
4. 2014
5. 2013
6. 2012

9. If you are able, please complete the volume of donated milk (L) for the following years:

Use "N/A" for the years the Milk Bank was not active or if the figures are not known.

1. 2017
2. 2016
3. 2015
4. 2014
5. 2013
6. 2012

10. If you are able, please complete the volume of donor milk (L) processed, issued and discarded during the following years:

Use "N/A" for the years the Milk Bank was not active or if the figures are not known.

1. 2018
2. 2017
3. 2016
4. 2015
5. 2014
6. 2013
7. 2012

11. **Is the donor allowed to donate from birth onwards?**
    - o Yes
    - o No

    If no, please specify from which postnatal week onwards

12. **Is the maximum duration of milk donation after delivery specified?**
    - o No
    - o Yes

    If yes, please specify (e.g. up to …… months after delivery)

13. **Please select which of the following are included in your donor screening process:**
    (Multiple answer selection is possible)
    - ☐ Smoking
    - ☐ Alcohol
    - ☐ Drugs of abuse
    - ☐ Medicines
    - ☐ HIV risk
    - ☐ extreme diets (e.g. vegans)
    - ☐ serological screening for Hepatitis B
    - ☐ serological screening for Hepatitis C
    - ☐ serological screening for HIV
    - ☐ serological screening for HTLV (human T cell leukemia virus)
    - ☐ serological screening for CMV (cytomegalovirus)
    - ☐ ALAT/ASAT (aspartate amino transferase/alanine amino transferase) ratio
    - ☐ Potential donors do not need to undergo a screening process
    - ☐ After travelling (region specific tests)

If donors are tested for more infections, please name the other infections tested

14. **Do donors receive instructions on how to express, store and handle the milk?**
    - o No
    - o Yes

If yes, please specify the type/format of instructions provided (e.g. written) and the service giving this information (e.g. a physician

15. **Handling at home: How long can breast milk be stored according to your advice before transferred to the milk bank?**
    1. Maximum duration of milk stored in the ***fridge*** **(before freezing)**
    2. Maximum duration of milk stored in the ***freezer***

16. **Do you have a home collection service?**
    - o Yes
    - o No

17. **Which are the acceptance criteria for donated milk?**

(Please tick all that apply)

- ☐ Proper labelling (time of milk expression and donor identification is clear)

☐ Evaluation of organoleptic characteristics (color and smell)
☐ The milk must be completely frozen
☐ The milk could be partially thawed

18. What is the maximum storage duration of raw donor milk before pasteurization at the milk bank?
If you don't pasteurize donor milk, please write down the maximum storage duration of raw donor milk.

1. In the refrigerator
2. In the freezer

19. How do you thaw the milk before applying the treatment?
In case no heat treatment (e.g. pasteurization, flash heating) is applied, please select thawing method(s) of raw donor milk.

(Please tick all that apply)
☐ Water bath
☐ Microwave
☐ Refrigerator
☐ Room temperature
☐ Orbital incubator
☐ Lukewarm running water
☐ Other (please specify)

20. Do you apply any heat treatment (e.g. pasteurization, flash heating) to donor milk?[2]
- ○ Yes
- ○ No

21. Do you perform pasteurization on donor milk?

1. **If yes, please describe the temperature (°C) and time that is used**
   (If you use an alternative method, please describe the method used).

2. **Describe pasteurizer design (e.g. shaking water bath, etc. If a specific equipment is used, name model and company if possible)**
   (If an alternative method is used, please describe treatment design.)

22. How long does the heating up to the required temperature (e.g. 62.5 °C) take?

23. How long does the cooling phase take and what temperature does the milk reach at the end of the cooling phase?
1. Duration
2. Temperature (°C)

[2] This question has a skip logic feature. If they answer YES, they continue to Q21 but if they answer NO, they go to Q28 (type of containers used). This was done to help out the banks that do not perform pasteurization (e.g. Norway) to complete the survey accordingly.

24. **Do you monitor the temperature during the whole process (e.g. a pasteurization cycle)?**
    - o No
    - o Yes

    If yes, please explain how

25. **Is the cooling phase automatically performed by the equipment (e.g. pasteurizer) used?**
    - o Yes
    - o No

If no, how is the milk chilled?

26. **Do you include the same volume of milk (ml) in every bottle within a pasteurization cycle?**
    1. If yes, please specify the volume used
    2. If no, please list all different examples of milk volumes used

27. **For how long and at which temperature is pasteurized donor milk stored?**
    1. Duration
    2. Temperature (°C)

28. **Type of containers used:**
    (Please tick all that apply)
    - ☐ Disposable
    - ☐ Reusable
    - ☐ Polypropylene
    - ☐ Polyethylene
    - ☐ Glass
    - ☐ Other (Please specify)

29. **What is the method of sterilization of the containers used?**
    - ☐ Ethylene oxide
    - ☐ Beta irradiation (or "e-beam")
    - ☐ Gamma irradiation
    - ☐ Other (please specify)

30. **Do you pool milk from several donors or only from a single donor?**
    If pooling is performed, please specify maximum number of donors

31. **Is all milk tested bacteriologically before pasteurization?**
    If donor milk is not pasteurized, please select all suitable answer options and use the comment box to explain.

    (Please tick all that apply)
    - ☐ Yes, every single container of donated milk
    - ☐ Yes, every sample of pooled milk
    - ☐ Only on first donation: A single container from the first donation
    - ☐ Only on first donation: All the pools of milk made from the first donation
    - ☐ Only occasionally
    - ☐ No, donor milk is not tested bacteriologically before pasteurization
    - ☐ Other (please specify)

32. **Do you perform microbiological analysis after pasteurization?**
If donor milk is not pasteurized, please use the comment box to explain.

(please tick all that apply)

- ☐ Always
- ☐ Only in a regular way (e.g. once a month or every 10 pasteurization cycles-please specify in the comment box below)
- ☐ Only when there are concerns about the processing
- ☐ Only on an ad-hoc basis if any new processes, equipment or staff are introduced
- ☐ Never
- ☐ Other (please specify)

33. **How do you perform the bacteriological testing?**
    1. Plating/ culture testing (please specify type of media used)
    2. Other (please specify)

34. **Which are the microbiological acceptance criteria of donor milk before and after pasteurization? please specify bacterial species and CFU/ml.**

Please use "10 to the power of (number) CFU/ml" to indicate bacteria number.

1. Before pasteurization
2. After pasteurization

35. **Safety of the overall milk banking process (from donor recruitment to provision of donor milk to each recipient) is monitored by:**
(Please tick all that apply)

- ☐ Good manufacturing practice, good laboratory practice
- ☐ Tracking and tracing
- ☐ Hazard analysis and critical control point (HACCP)
