# Supplementary material for: “Donor milk banking: Improving the future”. A survey on the operation of the European donor human milk banks
Source: PLoS One. 2021 Aug 19;16(8):e0256435. doi: 10.1371/journal.pone.0256435 (PMC8376009; doi:10.1371/journal.pone.0256435)
Supplement: S3 File — (PDF) [file pone.0256435.s003.pdf]

S3 File. Postnatal week onwards that the donors are allowed to donate, and the maximum duration of donation.

		Responses	n (%)
Donation	Start of donation	From birth onwards	92 (75)
		2-4 weeks	21 (17)
		≥ a month	7 (6)
		Other	3 (2)
	Maximum duration of donation	Not specified	44 (36)
		3 months	11 (9)
		4-5 months	2 (3)
		6 months	32 (26)
		>6-8 months	4 (3)
		9 months	3 (2)
		12 months	19 (15)
		Other	7 (6)
