# Supplementary material for: “Donor milk banking: Improving the future”. A survey on the operation of the European donor human milk banks
Source: PLoS One. 2021 Aug 19;16(8):e0256435. doi: 10.1371/journal.pone.0256435 (PMC8376009; doi:10.1371/journal.pone.0256435)
Supplement: S4 File — (PDF) [file pone.0256435.s004.pdf]

S4 File. Effect of frozen storage (-20°C and -80°C) on DHM components, before and after pasteurization.

		No loss	Decreased	Increased
Before pasteurization	-20°C, 1 months	Lactoferrin[21]	Lysozyme, sIgA, Lactoperoxidase[21]	
	-20°C, 3 months	Lactoferrin, lysozyme, IgG, IgA, C3[22]<br><br>Vitamin E, vitamin C, fatty acids[14]<br><br>Bactericidal activity against *P.aeruginosa*[15]<br><br>Lysozyme, Protease, lipase, B vitamins, lipids[12]	Lactoferrin (↓ 37%)[18], (↓ 55%)[13]<br><br>Bactericidal activity against *E.coli*[15]<br><br>Lactoperoxidase activity[12]<br><br>Fat content[16]	Dornic Acidity[23]
	-20°C, 5 months	Vitamin E, vitamin C, fatty acids[14]		
	-20°C, 6 months	IgA, EGF, IL-8, TGF-β2, TGF-β1, TNF-RI, TNF-α, IL-6, IL-10[17]	Lactoferrin (↓ 46%)[18], (↓ 65%)[18]<br><br>Bioactivity of lactoferrin[13]	
	-20°C, 8 months	Vitamin E, fatty acids[14]	Vitamin C[14]	
	-20°C, 9 months	Total protein, fat, lactoferrin, sIgA, osmolality[19]		Nonesterified fatty acids[14]
	-20°C, 12 months	Vitamin E, fatty acids[14]		
	-80°C, 3 months	Vitamin E[14]<br><br>Bactericidal activity against P aeruginosa, E. coli[15]		
	-80°C, 5 months	Vitamin E, vitamin C, fatty acids[14]		
	-80°C, 6 months	IgA, EGF, IL-8, TGF-β2, TGF-β1, TNF-RI, TNF-α, IL-6, IL-10[17]		
	-80°C, 8 months	Vitamin E, vitamin C, fatty acids[14]		
	-80°C, 12 months	Vitamin E, fatty acids[14]	Vitamin C[14]<br><br>IgA, IL-8,TGF-β1[17]	
After pasteurization	-20°C, 3 months	Dornic Acidity[23]		
	-20°C, 8 months	Macronutrient and energy content[20]		
