# Supplementary material for: “Donor milk banking: Improving the future”. A survey on the operation of the European donor human milk banks
Source: PLoS One. 2021 Aug 19;16(8):e0256435. doi: 10.1371/journal.pone.0256435 (PMC8376009; doi:10.1371/journal.pone.0256435)
Supplement: S5 File — (PDF) [file pone.0256435.s005.pdf]

S5 File. Published guidelines on storage, thawing, pooling and frequency of bacteriological testing of DHM.

Published guidelines	Freezing storage duration	Thawing	Pooling	Frequency of bacteriological screening
Recommendations for the creation and operation of maternal milk banks in Spain[26]	It is recommended that frozen milk not be kept at home for more than 4 weeks before submitting it to the HMB. Raw milk can be stored in the freezer before pasteurization at -20/-30°C up to 3 months or at -80°C up to 12 months from the date of extraction.	At 4°C prior to pasteurization, although partial thawing in a water bath is also allowed	HMBs can accept pooled raw milk extracted from a single donor.<br><br>Depending on the criteria established by each HMB, a mixture of acceptable raw DHM from different donors can be accepted as long as it is later pasteurized.	Before processing, each lot of milk will undergo testing according to the protocols validated by each HMB.<br><br>The microbiological contents of each lot of milk must be assessed after pasteurization.
Guidelines for the use of human milk and milk handling in Sweden[25]	Breast milk can be kept in a freezer (at least -20°C) for a maximum of 6 months	At room temperature, in a water bath with cold water fresh from the tap. This should take about 2-3 h for 100-200 ml. For larger volumes, defrost overnight in a refrigerator. Defrosted milk can be kept at room temperature for a maximum of 2 hours	Pooling of milk cannot be recommended	During continued milk donation, bacterial testing shall be performed once a month as a donor hygiene check.<br><br>For further control after pasteurization procedure systematic random sample controls (process control according to standard ISO 2859-1) may be performed, but there are no strong reasons for such a recommendation
European Milk Bank Association (EMBA)[1]	Not mentioned	In a refrigerator to prevent its temperature rising above 8°C or if impractical, because of time constraints, on a counter where it can be monitored and	The pooling of DHM from the same donor is acceptable prior to any heat processing	All pools of milk be tested prior to pasteurization.

		transferred to a refrigerator immediately once thawed.		Each batch of milk be tested after pasteurization
Guidelines for the establishment and operation of a donor human milk bank (Italy)[4]	Both fresh and pasteurized milk should be stored in the freezer at a temperature of −20°C. The maximum duration of storage in a freezer should be 6 months after the date of expression	Slowly in the refrigerator for a max period of 24 h, or rapidly in a water bath at a temperature not exceeding 37°C, or under running lukewarm water.	Pasteurization can be carried out on the milk of a single donor or on a milk pool deriving from a limited number of donors	Pre-pasteurization testing: at the first donation, and when the donor does not seem to guarantee the appropriate hygienic conditions or, in any case, periodically in a random manner<br>Post-pasteurization testing: in a regular way (e.g. once a month, or every 10 pasteurization cycles), or when there are concerns about the processing.
National Institute for Health and Care Excellence (NICE)[5]	Store donor milk awaiting pasteurization in the freezer at the milk bank (at −20°C) for no longer than 3 months from the date of expression.	Before testing and pasteurizing, thoroughly thaw the donor milk, and keep in the refrigerator for no longer than 24 h. Prevent the donor milk from reaching 8°C while thawing.	Only pool pre-pasteurized breast milk from the same donor. Do not pool milk from different donors, or batches of pasteurized breast milk from the same donor.	Before pasteurization, test a sample from each batch of pooled donor milk. Regularly test pasteurized donor milk for microbial contamination. Test either at least once a month or every 10 cycles, depending on which comes first and, on an ad,-hoc basis if any new processes, equipment or staff are introduced, or if there are concerns about any part of the process.
Guidelines for the construction and operation of a donor human milk bank (Austria)[28]	Frozen milk must be delivered to the milk bank within 3 months.<br>DHM can stay frozen for up to 6 months at −18°C or colder	Refrigerator (24 h), under running cold water or with special thawing devices.	Donor milk from various donors may be combined	A sample must be taken before each initial donation and also if there is any suspicion of contamination or non-hygienic handling practices throughout the donation process.

Guidelines for the organization of a donor human milk bank (Switzerland)[29]	Not specified	Refrigerator overnight or under running cold to lukewarm water	Not recommended	On three consecutive days at the beginning of the donation before pasteurization. As the donation progresses, a bacteriological check is carried out at least once a week. Routine bacteriological control is not necessary after pasteurization.
Guidelines for the operation and organization of a donor human milk (Norway)[27]	Breast milk can be kept in a freezer (-20°C) up to 6 months	The milk should be thawed in the refrigerator if possible. If there's not enough time, the milk can be thawed in a water bath (cold water) or at room temperature.	If DHM is dispensed raw, a mix form several donors should not be performed. If DHM is pasteurized a pool of usually not more than 5 donors is acceptable (Version 2002).<br><br>It is not recommended to mix DHM from different donors before pasteurization (Version 2018).	On first three donations and if the acceptance criteria are not met, testing is repeated for the next two donations. If these donations are rejected, the donation should be terminated
