# Supplementary figures and images for: “Donor milk banking: Improving the future”. A survey on the operation of the European donor human milk banks
Source: PLoS One. 2021 Aug 19;16(8):e0256435. doi: 10.1371/journal.pone.0256435 (PMC8376009; doi:10.1371/journal.pone.0256435)

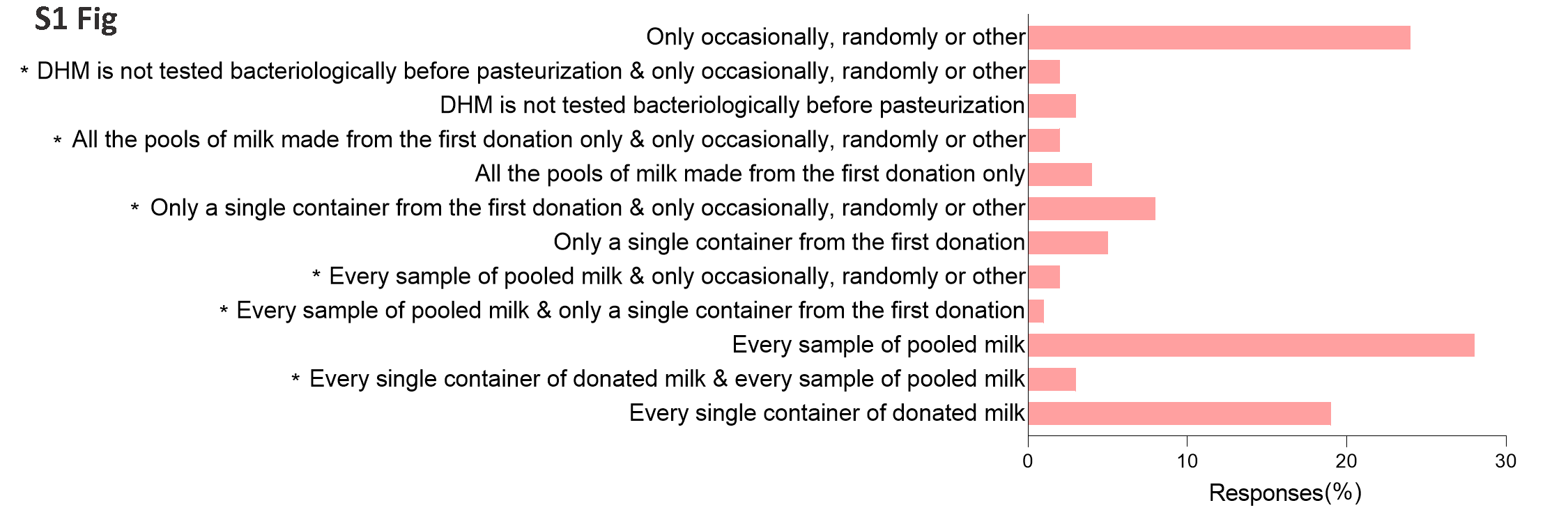

Supplement: S1 Fig — Multiple selection of answer options was possible; For all HMBs that selected more than 1 option, combined categories were created. *Combined categories. (TIF) [file pone.0256435.s006.tif]
